# Evaluation of M_x_O_y_/fucoidan hybrid system and their application in lipase immobilization process

**DOI:** 10.1038/s41598-022-11319-0

**Published:** 2022-05-04

**Authors:** Agnieszka Kołodziejczak-Radzimska, Michał Bielejewski, Andrzej Biadasz, Teofil Jesionowski

**Affiliations:** 1 Institute of Chemical Technology and Engineering, Faculty of Chemical Technology, Poznan University of Technology, Berdychowo 4, 60-965 Poznan, Poland; 2 Institute of Molecular Physics Polish Academy of Sciences, M. Smoluchowskiego 17, 60-179 Poznan, Poland; 3 Technical Physics, Faculty of Materials Engineering and Technical Physics, Poznan University of Technology, Piotrowo 3, 60-965 Poznan, Poland

**Keywords:** Biochemistry, Biotechnology, Materials science

## Abstract

In this work, new M_x_O_y_/fucoidan hybrid systems were fabricated and applied in lipase immobilization. Magnesium (MgO) and zirconium (ZrO_2_) oxides were used as M_x_O_y_ inorganic matrices. In the first step, the proposed oxides were functionalized with fucoidan from *Fucus vesiculosus* (Fuc). The obtained MgO/Fuc and ZrO_2_/Fuc hybrids were characterized by means of spectroscopic analyses, including Fourier transform infrared spectroscopy, X-ray photoelectron spectroscopy, and nuclear magnetic resonance. Additionally, thermogravimetric analysis was performed to determine the thermal stability of the hybrids. Based on the results, the mechanism of interaction between the oxide supports and fucoidan was also determined. Furthermore, the fabricated M_x_O_y_/fucoidan hybrid materials were used as supports for the immobilization of lipase from *Aspergillus niger*, and a model reaction (transformation of *p*-nitrophenyl palmitate to *p*-nitrophenol) was performed to determine the catalytic activity of the proposed biocatalytic system. In that reaction, the immobilized lipase exhibited high apparent and specific activity (145.5 U/g_catalyst_ and 1.58 U/mg_enzyme_ for lipase immobilized on MgO/Fuc; 144.0 U/g_catalyst_ and 2.03 U/mg_enzyme_ for lipase immobilized on ZrO_2_/Fuc). The immobilization efficiency was also confirmed using spectroscopic analyses (FTIR and XPS) and confocal microscopy.

## Introduction

In recent decades, development of inexpensive, biodegradable, and easily available natural materials in different application have been of more interest for the huge number of researchers. It is highly preferable that the carrier matrix that binds the enzyme can be produced reproducibly and does not disrupt the enzyme activity, because it has a great importance for technological performance and commercial success^1–3^. However, those materials also have some disadvantages (low mechanical strength and limited thermal stability) which can be improved using appropriate modification process^4,5^.

Biopolymers, due to their versatile properties, including non-toxicity, biocompatibility, biodegradability, flexibility, and renewability, are promising carriers for enzyme immobilization^6–8^. In addition, numerous reactive functional groups, such as hydroxyl, amino, or carboxylic acid groups, are present in their chemical structure. These allow enzymes to connect with that structure^7,9^. To date, various natural polysaccharides such as cellulose^10,11^, chitin^12,13^, chitosan^14,15^, alginate^16,17^, agarose^18–20^ and carrageenan^21,22^ have been used as enzyme supports. Recently, more attention has been paid to biopolymer/inorganic matrix composites or hybrids, which can also be used in enzyme immobilization^7^. High resistance, stability, and availability are the most important parameters of inorganic materials, especially selected oxides (SiO_2_, ZnO, ZrO_2_, MgO, etc.)^23^. Moreover, they can be synthesized via simple and fast methods, which makes them relatively cheap. It should also be noted that biopolymers can be introduced on the surface of metal oxides to increase their affinity to enzymes^7,24^. There is a great deal of information in the literature concerning materials based on chitosan/chitin/cellulose and inorganic oxides, and their application in enzyme immobilization^25–28^.

Natural-derived polysaccharides play an important role in the pharmaceutical and cosmetics industries. They are widely obtained from algae, including brown algae^29,30^. Brown algae (*Phaeophyta*) are a group of algae with a very high degree of specialization in the structure of the thallus, which most often has the form of a branched thread^31–33^. Seaweed is a source of potentially bioactive polysaccharides, among which fucoidan extracted from brown seaweed (especially from *Fucus* species and tissues of echinoderms) is currently the compound being studied most extensively^34,35^. Fucoidan can be obtained from a number of marine sources, including sea cucumbers^36^ and brown algae^37^. A high fucoidan content has been established in a large number of algae and invertebrates, for example, *Fucus vesiculosus*, *Sargassum stenophyllum*, *Chorda filum*, *Ascophyllum nodosum*, *Dictyota menstrualis*, *Fucus evanescens, Fucus serratus*, *Fucus distichus*, *Caulerpa racemosa*, *Hizikia fusiforme*, *Padina gymnospora, Kjellmaniella crassifolia, Analipus japonicus,* and *Laminaria hyperborea*. Different types of fucoidan are present in these sources, and various extraction methods are used to obtain them^38^.

Fucoidan consists of α-l-fucopyranose molecules, which may be connected by 1→3 bonds or by alternating 1→3 and 1→4 bonds. To obtain the branched structure in the main chain, radicals of α-l-fucopyranose are attached, as well as inorganic sulfate(VI) radicals and radicals of organic such as d-glucuronic and acetyl. Several of the fucoidan structures have also contain small amounts of various other monosaccharides e.g. glucose, galactose, xylose, and/or mannose^39–42^. Fucoidan offers a variety of biological properties, including antibacterial, antioxidant, antiviral, anti-inflammatory, anticoagulant, and anticancer properties^43–45^. In addition, fucoidan is biocompatible, biodegradable, and nontoxic^46,47^.

Due to its unique features, fucoidan is a promising biopolymer that can also be utilized as a coating material. To date, fucoidan-modified materials have been used in medical applications such as drug delivery^48–52^. The magnetic mesoporous silica system was modified with fucoidan by Moorthy et al.^48^. Fucoidan was applied to the silica surface by a metal–ligand complex coordination technique. The proposed material was utilized as a drug carrier and as a hyperthermia agent for chemotherapy and magnetic hyperthermia-based thermal therapy applications in emerging cancer therapy. In other studies, fucoidan was coated on CuS nanoparticles and applied in chemophotothermal therapy against cancer cells^49^. In this case, fucoidan was introduced onto the CuS surface by a layer-by-layer technique using polycationic and anionic compounds. In another study, Shin et al.^50^ developed fucoidan-coated manganese dioxide nanoparticles (Fuco-MnO_2_-NPs) and tested them in the clinical treatment of cancer. The MnO_2_-NPs were coated with fucoidan by the adsorption method. Venkatesan et al.^51^ used a fucoidan–chitosan complex to modify silver nanoparticles (AgNP). This system has high potential for food and cosmetic applications. Another research team designed bimodal fucoidan-coated zinc oxide/iron oxide nanoparticles for medical applications^52^.

Until now, inorganic materials modified with fucoidan have not been investigated as supports for enzyme immobilization. Therefore, in this study, selected inorganic M_x_O_y_ compounds (ZrO_2_ and MgO) were modified with fucoidan (Fuc). A key objective was to confirm the modification of M_x_O_y_ with fucoidan using a range of spectroscopic analyses—Fourier transform infrared spectroscopy (FTIR), nuclear magnetic resonance spectroscopy (NMR), and X-ray photoelectron spectroscopy (XPS). Additionally, thermogravimetric analysis (TG/DTG) was performed. The prepared M_x_O_y_/Fuc materials were then used as supports for lipase. The amount of immobilized lipase, catalytic activity, and confocal microscopic images were analyzed to confirm the success of the immobilization.

## Experimental part

### Materials

The following materials were used in the study: zirconium isopropoxide (TPZ), 25% ammonia solution (NH_3aq._), ethyl alcohol (EtOH), magnesium oxide powder, fucoidan from *Fucus vesiculosus* (Fuc), lipase from *Aspergillus niger* (LAN), sodium phosphate (NaH_2_PO_4_), dibasic sodium phosphate (Na_2_HPO_4_) *p*-nitrophenyl palmitate (*p*-NPP), *p*-nitrophenol (*p*-NP), 2-propanol, Triton X-100 and Arabic gum. All of these materials were purchased from Sigma-Aldrich (Saint Louis, MO, USA).

### M_x_O_y_/Fuc hybrid preparation

The high-purity magnesium oxide powder (98%) used in these studies was purchased from Sigma-Aldrich. ZrO_2_ was synthesized using the sol–gel method. In this case, zirconium isopropoxide and ammonium were dosed into a reactor containing ethanol. The mixture was stirred for 1 h. The next step of the synthesis was crystallization (ageing; 24 h). The resulting zirconium oxide (ZrO_2_) was washed with water and filtered, and then dried at 105 °C.

At the next stage of the research, ZrO_2_ and MgO were modified with fucoidan. For this purpose the water solution of fucoidan (1 mg/mL) was prepared, 1 g of the appropriate oxide was added to the fucoidan solution (10 mL), and it was stirred magnetically for 24 h. The resulting M_x_O_y_/Fuc hybrid system was filtered and dried at 60 °C. The samples obtained were labeled ZrO_2_/Fuc and MgO/Fuc.

### Spectroscopic analysis

To confirm both the functionalization of ZrO_2_ and MgO with fucoidan and the immobilization process, spectroscopic analyses were performed: Fourier transform infrared spectroscopy (FTIR), nuclear magnetic resonance (^1^H and ^13^C NMR) and X-ray photoelectron spectroscopy (XPS). A detailed description of these analyses is given in the Supplementary Materials.

### Thermogravimetric analysis

Thermogravimetric analysis (TG/DTG) was performed using a Jupiter STA 449F3 thermogravimetric analyzer (Netzsch, Germany). Measurements were carried out under flowing nitrogen at a heating rate of 5 °C/min and in a temperature range of 30 to 1000 °C, with an initial sample weight of approximately 5 mg.

### Enzyme immobilization

ZrO_2_/Fuc and MgO/Fuc were used as supports for lipase immobilization. The process was performed by an adsorption method. A specified amount of matrix was shaken with the lipase solution (5 mg/mL, phosphate buffer at pH = 7) in an incubator (20 °C, IKA-Werke, Staufen, Germany) for 24 h. Then the prepared biocatalytic system (ZrO_2_/Fuc/LAN or MgO/Fuc/LAN) was separated by filtration. Bradford analysis^53^ was used to confirm the result of the immobilization process and to calculate the amount of immobilized lipase (*P*_*LAN*_, mg_enzyme_/g_support_) and the immobilization performance (PI,%). In the next step, the enzymatic activity of the immobilized lipase was evaluated. The model reaction used was the transformation of *p*-NPP (*p*-nitrophenyl palmitate) to *p*-NP (p-nitrophenol). The release of the product was observed at 410 nm (using a JASCO V650 spectrophotometer, Japan). All reactions (performed in triplicate) were carried out with stirring at 1000 rpm for 2 min at 30 °C. Based on the results, the apparent (U/g_catalyst_), specific (U/mg_enzyme_), and relative (%) activities were estimated. The equations used to calculate activity are given in the Supplementary Materials. Kinetic parameters, including the Michaelis–Menten constant (K_M_) and maximum reaction velocity (V_max_) were determined by means of an enzymatic assay based on the same reaction mentioned above, using various concentrations of the substrate solution (0.005–1.5 M). The apparent kinetic parameters of the free and immobilized enzyme were calculated based on the Hanes–Woolf plot. The immobilization efficiency was also indirectly confirmed by FTIR and XPS analysis.

### Confocal microscopy

The morphology of the M_x_O_y_/Fuc hybrids and immobilized lipase was evaluated on the basis of confocal laser scanning microscopy (CLSM) photographs (LSM710, Zeiss, Germany), obtained using an argon laser (488 nm). In material mode (reflected light), the laser operated at a wavelength of 458 nm. In fluorescence mode, the laser operated at 488 nm and fluorescence was observed in the range 510–797 nm.

## Results and discussion

### Spectroscopic analysis of ZrO_2_/Fuc and MgO/Fuc hybrids

**Figure 1:**
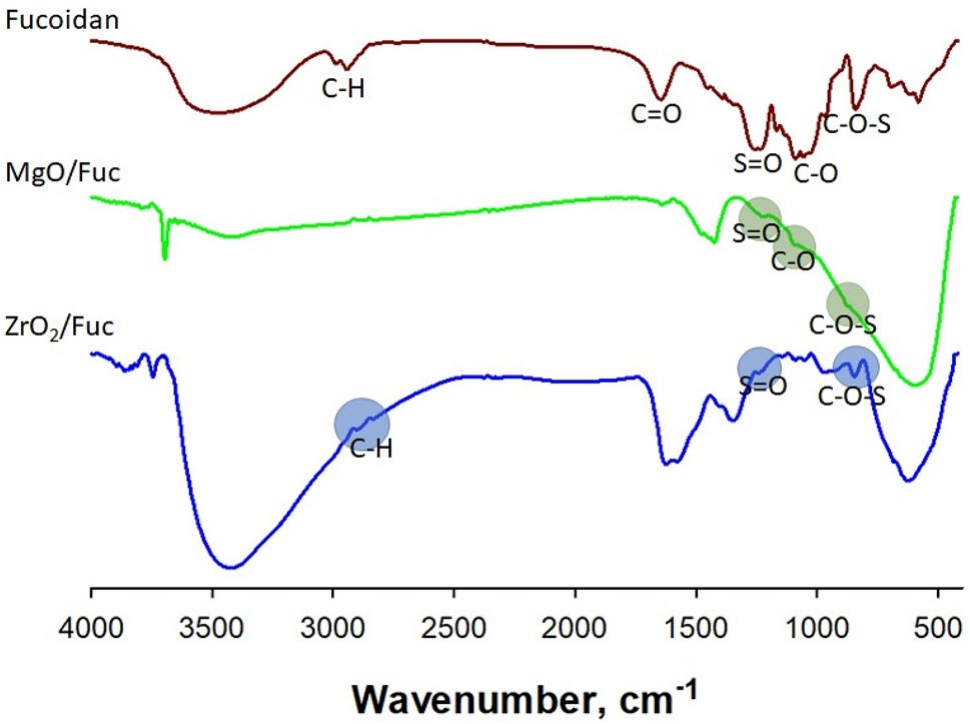
FTIR spectra of fucoidan, MgO/Fuc, and ZrO_2_/Fuc.

Spectroscopic analysis is employed to explore hybrid materials, providing practical information such as elemental type, chemical composition, optical and electronic properties, and crystallinity. In this study, spectroscopic analyses were used to confirm the functionalization of ZrO_2_ and MgO with fucoidan. The characteristic groups in the fucoidan structure were precisely interpreted based on the FTIR spectrum (Fig. 1). The following bands were identified: OH group of monosaccharide monomer (at 3500 cm^−1^); aliphatic C–H (at 2983 and 2945 cm^−1^); O–C–O stretching vibrations (at 1635 cm^−1^); asymmetric stretching vibrations of S=O in the sulfate group (at 1258 cm^−1^); ether bond C–O (at 1070 cm^−1^); C–O–S (at 846 cm^−1^); and a characteristic band for deoxy sugars such as fucose (at 572 cm^−1^). Additionally, a signal at 846 cm^−1^ corresponds to sulfation in the equatorial position, where the sulfate ester binds to the C-2 of fucose to form sulfate fucose^54,55^.

The FTIR spectra of MgO and ZrO_2_ modified with fucoidan are presented in Fig. 1. They confirm that the metal oxide was successfully functionalized with fucoidan. The characteristic groups of pure oxide (Fig. 1S) and fucoidan appear on the FTIR spectra of MgO/Fuc and ZrO_2_/Fuc. On the FTIR spectrum of MgO/Fuc (Fig. 1) the following fucoidan groups are present: OH (at 3450 cm^−1^); C=O (at 1650 cm^−1^); S=O (at 1450 cm^−1^); C–O (at 1150 cm^−1^). The small band for C–O groups and the small and broad band for OH groups indicate the direct connection of the fucoidan with the MgO surface.

The FTIR spectrum for ZrO_2_/Fuc contains bands for OH (at 3450 cm^−1^); C–H (at 2900 cm^−1^); S=O (1400 cm^−1^); and C–O–S (at 800 cm^−1^) (Fig. 1). The broad and intensive band with a maximum at 3500 cm^−1^ indicates the connection of fucoidan with ZrO_2_ via hydrogen bonds of water molecules. This is further evidenced by the shift in this band relative to the band obtained for pure fucoidan. The narrow and weak peak at 3700 cm^−1^ may originate from pure water, which may attach to the fucoidan-modified surface of ZrO_2_ during the fucoidan functionalization process in aqueous solution.

**Figure 2:**
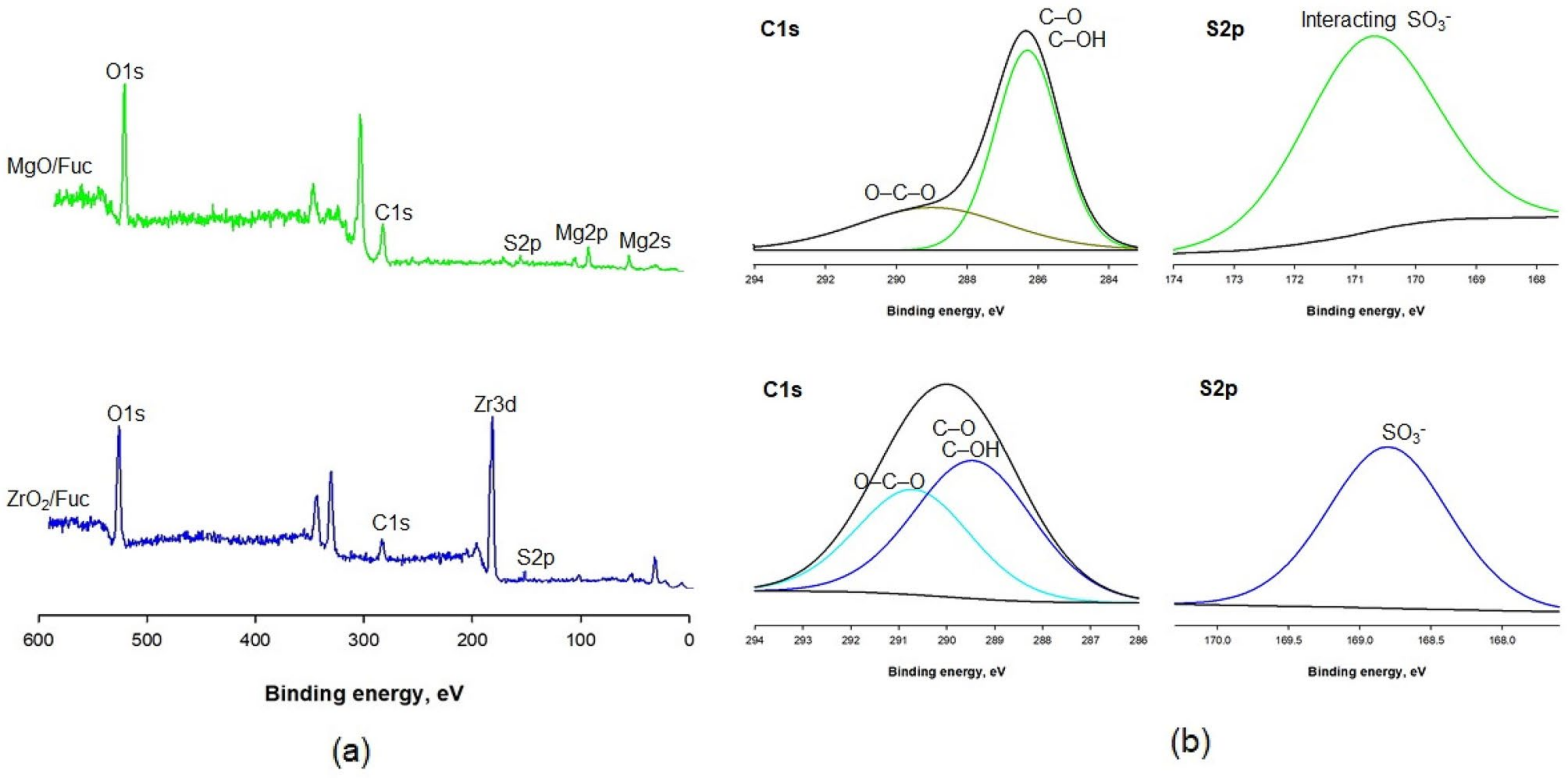
XPS survey spectra of MgO/Fuc and ZrO_2_/Fuc (**a**), and the deconvoluted XPS of C 1s for MgO/Fuc; S 2p for MgO/Fuc; C 1s for ZrO_2_/Fuc; and S 2p for ZrO_2_/Fuc (**b**).

XPS spectroscopy was also used to confirm the functionalization of MgO and ZrO_2_ with fucoidan. The results are shown in Fig. 2. On the surface of MgO and ZrO_2_ (Fig. 2S), elements such as magnesium, zirconium, and oxygen are present. The MgO/Fuc and ZrO_2_/Fuc hybrids contain the same elements, but carbon and sulfur are also present, which is a consequence of the modification with fucoidan (Fig. 2a).

Additional information on the interaction of fucoidan with the surface of the inorganic oxide is provided by a detailed analysis of the C 1s and S 2p lines of the XPS spectrum. The XPS C 1s and S 2p lines of the MgO/Fuc and ZrO_2_/Fuc hybrids are shown in Fig. 2b. Carbon and sulfur atoms are not present on the surface of MgO and ZrO_2_ (Fig. 2S). In the XPS C 1s line for MgO/Fuc, the two C1s peaks were identified at around 288.7 eV and 286.2 eV, corresponding respectively to O–C–O and C–OH/C–O bonds. Here, the C–OH/C–O bonds are more expanded. A different situation is observed with the C 1s line for ZrO_2_/Fuc. In this case, the two C 1s peaks are also recognized at around 290.8 eV (O–C–O) and 289.4 eV (C–OH/C–O). However, the size of these peaks is similar. Deconvolution of the fucoidan S 2p spectral peak shows two sulfur chemical environments at approximately 167.0 eV (for the –SO_3_^–^ group) and 170.9 eV (for the interacting –OSO_3_^–^ group)^56–58^. For MgO/Fuc and ZrO_2_/Fuc, only one sulfur environment was observed, at binding energies of 170.7 eV (interacting –OSO_3_^–^ group) and 168.8 eV (–SO_3_^–^ group) respectively (Fig. 2b). The change in the binding energy peak may indicate interactions of some fucoidan sulfate groups with the surface of the metal oxide^58^.

**Figure 3:**
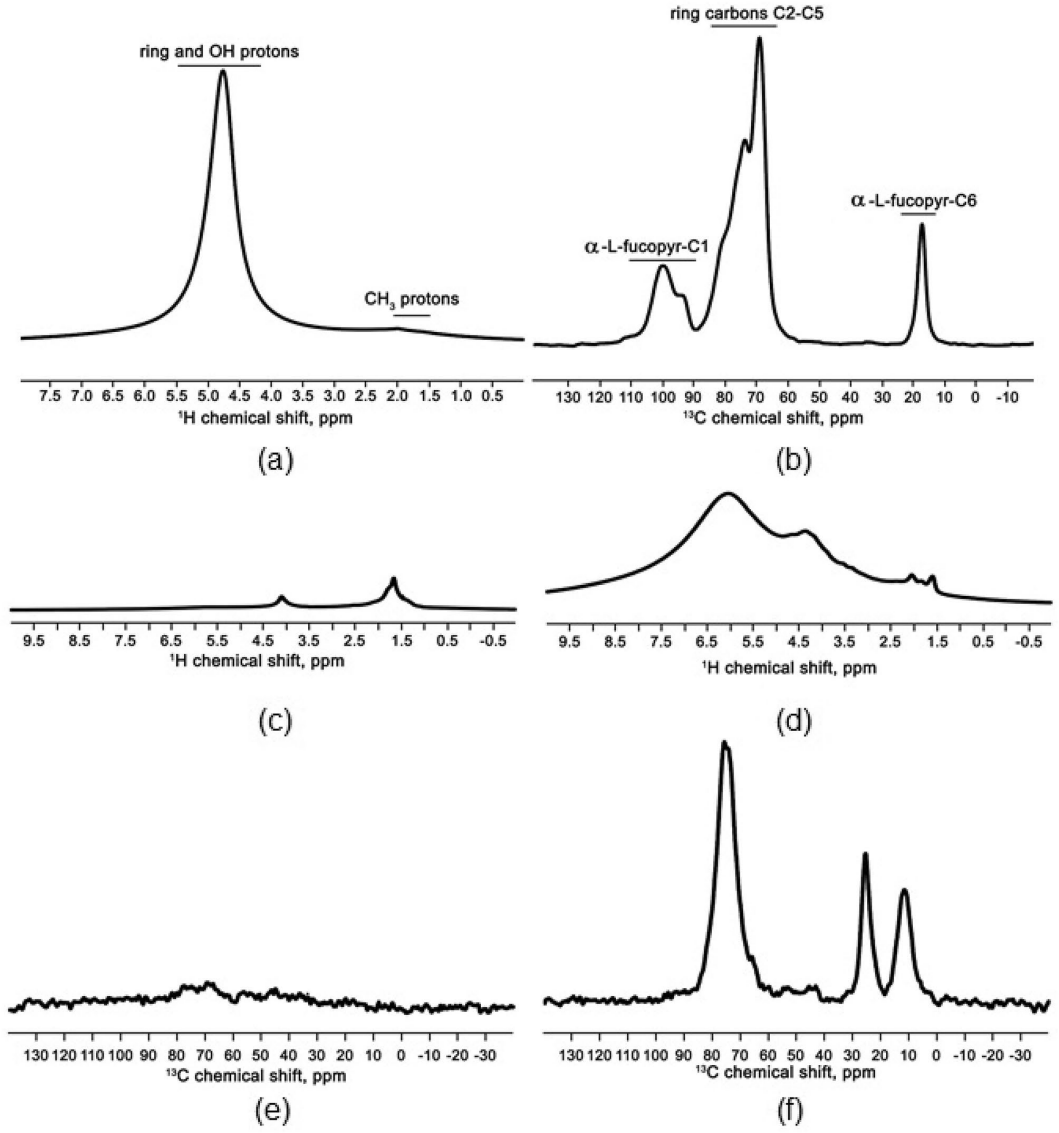
Solid-state DP/MAS SE ^1^H NMR spectrum (**a**) and CP/MAS ^13^C NMR spectrum (**b**) of fucoidan at room temperature. Solid-state DP/MAS ^1^H NMR spectra for MgO/Fuc (**c**) and ZrO_2_/Fuc (**d**) at room temperature; and solid-state CP/MAS ^13^C NMR spectra for MgO/Fuc (**e**) and ZrO_2_/Fuc (**f**) at room temperature.

The ^1^H and ^13^C spectra of pure fucoidan (Fig. 3) provide us with information about the structure of the pure polysaccharide and the characteristic hydrogen bond pattern of the material used, and serve as a reference for the analysis of the data obtained for the investigated samples. Moreover, since the samples were obtained by wet synthesis, a signal from residual water molecules was expected to be found in the ^1^H spectra of the functionalized matrices. ^13^C spectra were used to confirm that fucoidan molecules were present in the matrices. Differences in ^13^C spectra between the neat fucoidan sample and the functionalized one will indicate a change in the structure of the polysaccharide chain caused by interaction with the surface of the matrix. As fucoidan molecules can form hydrogen bonds, the change in the ^1^H spectra would indicate a mechanism of functionalization of the surface of the matrices with fucoidan molecules, based on the creation of hydrogen bonds between the surface of the matrix and the fucoidan molecules.

The ^1^H spectrum (Fig. 3a) shows an intense signal in the region 5.5–4.25 ppm, which comes from ring protons bonded to C2–C5 carbons, OH groups of fucoidan and residual water molecules. Small signals at 2–1.5 ppm originate from methyl groups located at C6 carbons. The ^13^C spectrum (Fig. 3b) shows a strong signal at 16.8 ppm identified as C6 carbons in methyl groups, while the broad signal at 85–60 ppm comes from C2–C5 ring carbons, and the signal at 100 ppm from C1 carbons.

Figure 3c–f shows the ^1^H and ^13^C spectra recorded for matrices functionalized with fucoidan molecules. In the ZrO_2_/Fuc ^1^H spectrum a strong OH signal from fucoidan and residual water is observed (Fig. 3c). The observed change in the chemical shift of the most intense signal in the range 5.5–4.25 ppm is related to the OH groups in fucoidan and residual water, indicating that the structure of the hydrogen bonds present in the samples is changed. In the case of ZrO_2_ (Fig. 3c), a splitting of the signal in a range from 7.5 to 3.5 ppm can be observed. This splitting is caused by OH groups involved in the creation of hydrogen bonds between fucoidan molecules and the surface of the ZrO_2_ matrix. The peak shifts towards lower fields by 1.4 ppm, indicating the creation of strong hydrogen bonds. The second peak appearing at 4.3 ppm corresponds very well to proton signals from the ring unit of the fucoidan molecule. Together with the lack of change in the signal position at 2–1.5 ppm, this implies that the mechanisms of functionalization of the ZrO_2_ matrix with fucoidan are due to the creation of hydrogen bonds. The MgO matrix used was purchased commercially and used without further preparation for the functionalization procedure. The proton spectra of this matrix functionalized with fucoidan are shown in Fig. 3e. Only small signals from fucoidan molecules were detected, with no peak from residual water molecules. The observed chemical shifts correspond to those of fucoidan molecules found in the literature, and no shift of the hydroxyl group signal was detected.

Direct evidence of the presence of fucoidan molecules in the studied matrices is provided by the ^13^C spectra. The ^13^C spectra of the studied samples are shown in Fig. 3e,f. Only the ZrO_2_ matrix functionalized with fucoidan shows well-detectable signals from the fucoidan units; in the case of MgO matrices only very weak signals from aromatic carbons were detected. By comparing the ^1^H and ^13^C spectra of the studied samples we can draw conclusions about the chemical composition of the samples. The MgO matrix purchased commercially was found to be free of any impurities after the synthesis processes, but only a very small amount of fucoidan molecules was found in the functionalized matrix. The weak ^1^H signals from the fucoidan molecules indicate a very low quantity of this substance, which is also confirmed by the almost undetectable ^13^C signals from the fucose unit. Another important finding is that the signals from residual water molecules expected to be left after the functionalization process are also missing.

On the basis of the spectroscopic analyses it can be concluded that the functionalization of oxide materials with fucoidan molecules was successful. However, the NMR analysis shows that a better effect was achieved for ZrO_2_. Fucoidan molecules were shown to attach to the surface of the ZrO_2_ matrix by way of hydrogen bonds; the molecules may be attached directly or via residual water molecules. Furthermore, only a very small trace of fucoidan units was found after functionalization of MgO. Also, it is important to note that no signal from the hydroxyl groups of water molecules was detected in this sample. Therefore, it can be concluded that water plays an important role in the functionalization procedure and mediates the binding of fucoidan molecules to the surface of the matrix. In addition, FTIR and XPS analyzes confirm the positive modification with fucoidan, where the characteristic bonds/picks for fucoidan are observed.

### Thermogravimetric analysis of MgO/Fuc and ZrO_2_/Fuc

**Figure 4:**
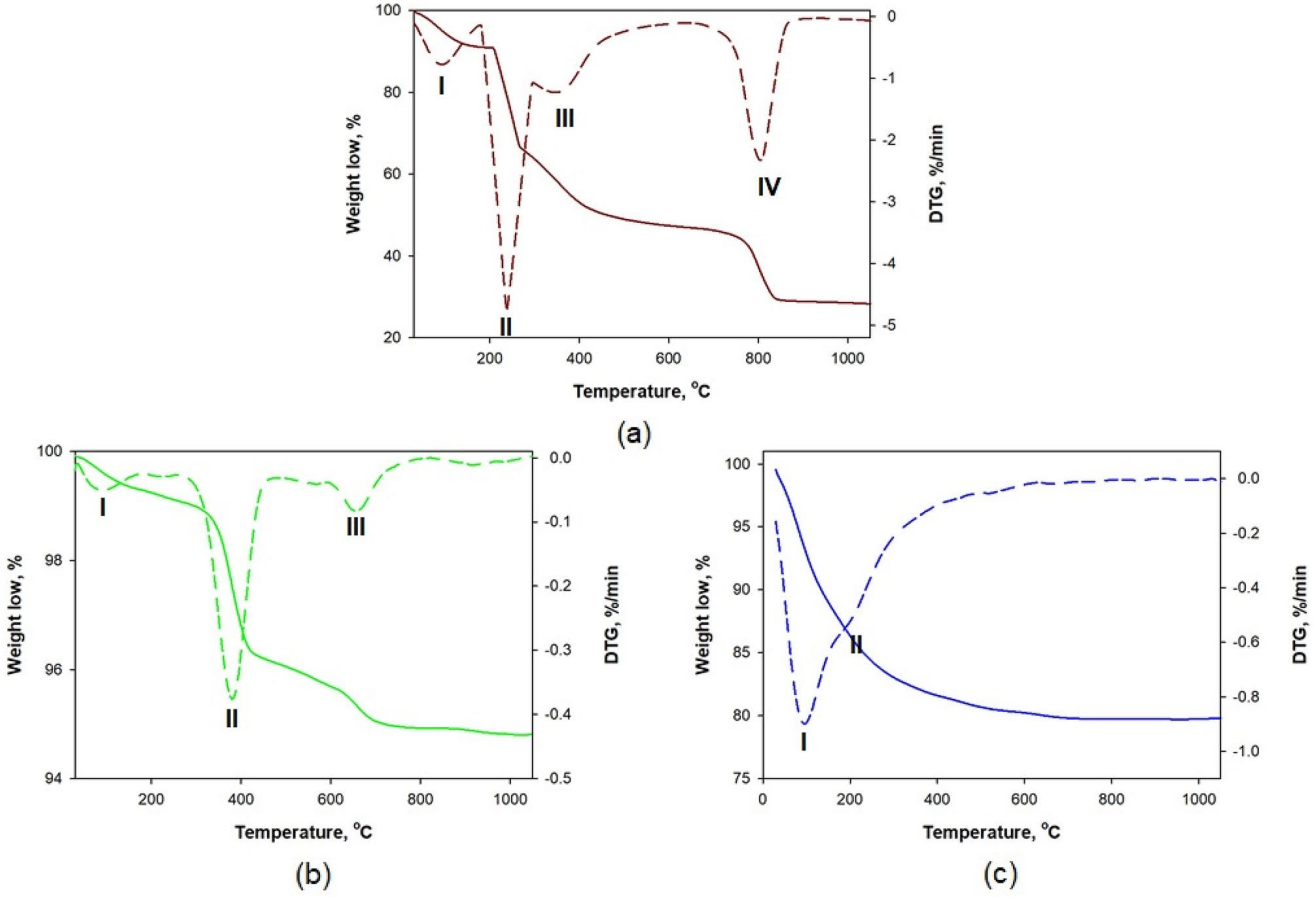
TG/DTG curves of fucoidan (**a**); MgO/Fuc (**b**); and ZrO_2_/Fuc (**c**).

Figure 4 shows the TG/DTG curves of fucoidan and the MgO/Fuc and ZrO_2_/Fuc hybrids. The fucoidan thermogram (Fig. 4a) presents four mass loss steps. The first mass loss (exothermic peak I) of 6% at 100 °C corresponds to physically adsorbed water. The second and third, at 240 °C (approximately 22%, exothermic peak II) and 355 °C (42%, exothermic peak III) are associated with the loss of sulfate groups. The final mass loss, of approx. 70%, occurring at 800 °C (exothermic peak IV) is probably related to the decomposition of carbon residues^48,59^. Furthermore, changes are observed between the TG/DTG curves of pure inorganic oxide (Fig. 6S) and the sample after fucoidan functionalization (Fig. 4b,c). The MgO/Fuc sample exhibited a total mass loss of 5%. The mass loss took place in three steps, with major losses in the second stage (ca. 4% at 380 °C) and in the third (5% at 650 °C), probably corresponding to the collective decomposition of the surface-coating fucoidan polymeric units^59^. A change in total mass loss is also observed on the TG/DTG curves of ZrO_2_ (Fig. 8S) and ZrO_2_/Fuc (Fig. 4c). In this case, the ZrO_2_/Fuc hybrid loses about 20% of its total mass. Here, only two exothermic peaks are observed (at 100 and 220 °C), associated with physically and chemically adsorbed water. The nature of the mass loss and the missing peak above 600 °C suggest that the fucoidan molecules evaporated from the system before the decomposition of carbon residues occurred. This can be understood if the fucoidan molecules were bonded to the ZrO_2_ surface via water molecules.

However, the changes in the total mass loss of both samples after fucoidan modification (MgO/Fuc and ZrO_2_/Fuc) suggest that fucoidan was successfully incorporated on the surface of MgO and ZrO_2_.

### Enzyme immobilization

**Figure 5:**
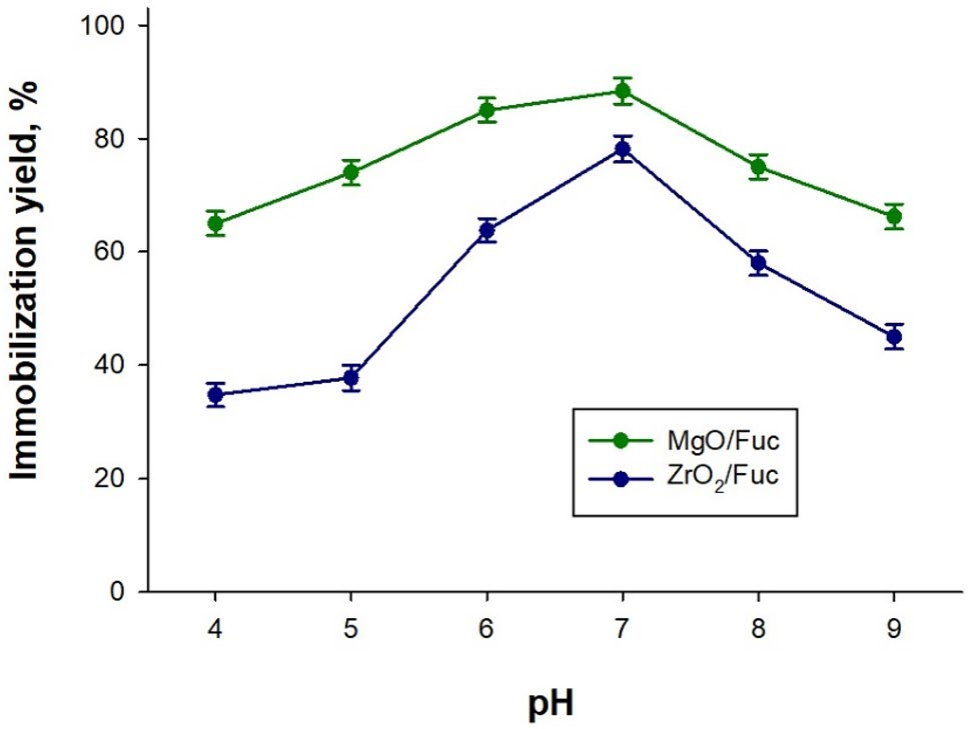
Effect of pH on the immobilization of lipase from *Aspergillus niger* on MgO/Fuc and ZrO_2_/Fuc.

The designed MgO/Fuc and ZrO_2_/Fuc hybrid materials were used as supports for lipase immobilization. The effect of pH on lipase immobilization was investigated as shown in Fig. 5. The immobilization yield lipase on MgO/Fuc increased from 65% at pH 4 to the highest value of 88.4% at pH 7.0, but decreased to 66.2% at pH 9. The immobilization yield of lipase on ZrO_2_/Fuc showed a similar tendency, which was 34.8% at pH 4, reaching the highest value of 78.2% at pH 8, and decreased to 45% at pH 9. The protons bind to the lone pair electrons on the amino N atom under acidic conditions. Whereas under pH 8, the hydrogen bonds on support will be broken in alkalinity conditions^4,5^. Based on the information, the optimal pH for immobilization was determined to be 7.0.

Basic information on enzyme immobilization efficiency and the catalytic activity of lipase immobilized on MgO/Fuc and ZrO_2_/Fuc is given in Table 1S. The results for catalytic parameters indicate that the materials proposed in this study (both MgO/Fuc and ZrO_2_/Fuc) can be used as supports for the enzyme. The amount of lipase immobilized was 91.8 mg on 1 g of MgO/Fuc, and 70.8 mg on 1 g of ZrO_2_/Fuc. The immobilization efficiencies were 61.2% and 78.6%, respectively. The obtained biocatalytic systems (MgO/Fuc/LAN and ZrO_2_/Fuc/LAN) exhibited similar catalytic activities. Lipase immobilized on MgO/Fuc had apparent and specific activities of 145.5 U/g_catalyst_ and 1.58 U/mg_enzyme_, while the corresponding values for the ZrO_2_/Fuc/LAN biocatalytic system were *A*_*Ap*_ = 144.0 U/g_catalyst_ and *A*_*S*_ = 2.03 U/mg_enzyme_.

**Figure 6:**
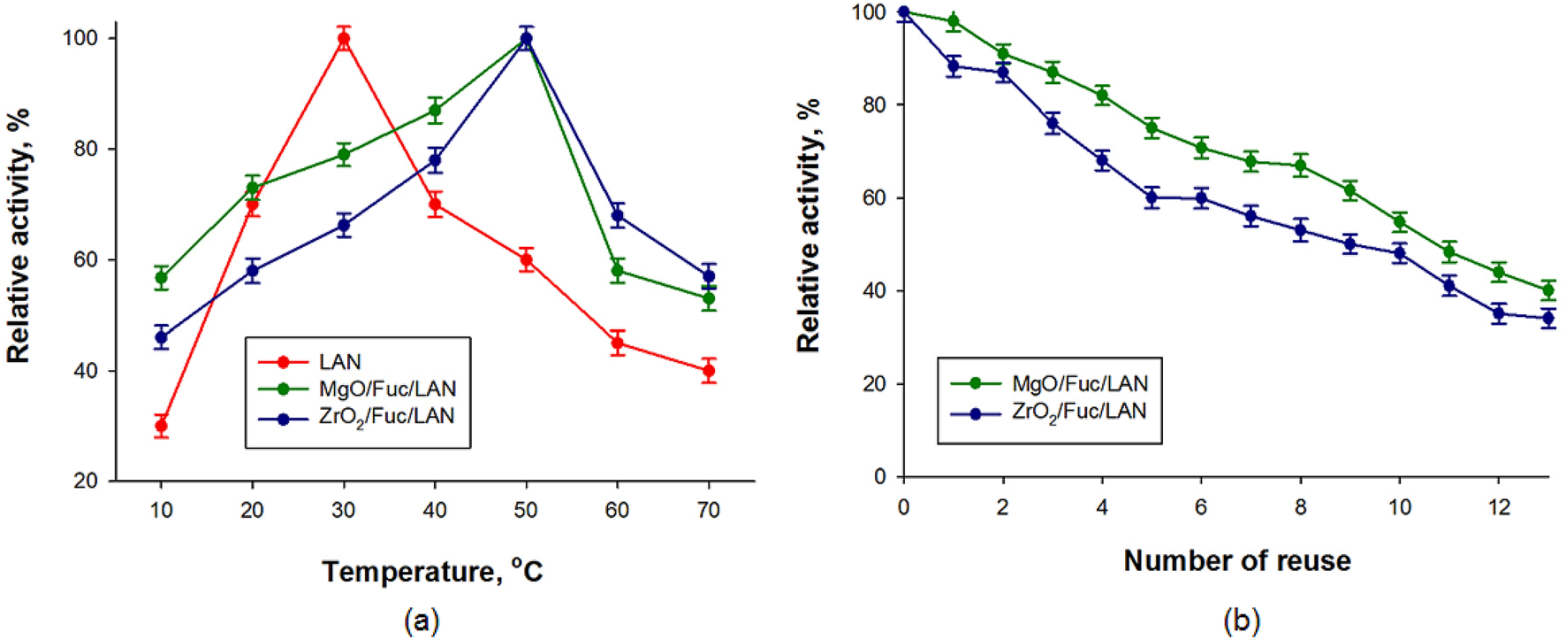
Influence of temperature (**a**) and repeated use on the catalytic activity of free and immobilized lipase (**b**).

The influence of temperature and reuse over several cycles on the enzymatic activity of the immobilized lipase was evaluated (Fig. 6). The results show that lipase immobilized on both MgO/Fuc and ZrO_2_/Fuc retained above 40% of its activity at temperatures in the range 20 to 70 °C (Fig. 6a). In both cases (MgO/Fuc/LAN and ZrO_2_/Fuc/LAN) the maximum activity was achieved at 50 °C, which indicates that lipase immobilized on the proposed materials can be used in harsher conditions. Moreover, the immobilized enzyme has a heterogeneous form and can be used over several enzymatic reaction cycles. The tests showed that the proposed biocatalytic systems (MgO/Fuc/LAN and ZrO_2_/Fuc/LAN) retained ca. 40% of their initial activity after 12 cycles (Fig. 6b).

In addition the kinetic parameters like Michaelis–Menten constant (K_M_) and maximum reaction velocity (V_max_) were determined. The obtained results are shown in Table 1. A lower K_M_ value after immobilization indicates a higher binding ability with respect to the substrate. While, the higher V_max_ suggesting that the immobilized lipase can catalyze the reaction faster than free lipase.

**Table 1:** Kinetic parameters (K_M_ and V_max_) of free and immobilized lipase from *Aspergillus niger.*

Sample	K_M_, mM	V_max_, mM/min
Free lipase from *Aspergillus niger*	0.58 ± 0.02	43.55 ± 0.6
Lipase immobilized on MgO/Fuc	0.68 ± 0.02	51.74 ± 0.7
Lipase immobilized on ZrO_2_/Fuc	0.61 ± 0.03	49.53 ± 0.7

Chitin, chitosan, and cellulose are the polysaccharides most commonly used to modify inorganic oxides. A comparison of the results obtained in this study with previous results on lipase immobilization on various polysaccharide-based hybrids is shown in Table 2. The results show that materials based on polysaccharide and inorganic oxide can be successfully applied as supports for the immobilization of lipase (of various origins). Different types of lipase (from *Candida rugosa*, *Porcine pancreas*, *Aspergillus niger* and *Rhizomucor miehei*) were immobilized on the following matrices: chitin/graphene oxide, chitosan/mesoporous silica, magnetic nanoparticles coated with chitosan, and cellulose/Fe_2_O_3_^60–63^. The immobilized lipases exhibited enzymatic activity in the range 125–328 U/g, and could be used over several reaction cycles (from 5 to 14), retaining 50–90% of their initial activity. The results obtained in the present study are similar to others; the observed difference is probably due to the use of different kinds of lipases, which exhibit different enzymatic activities in their native form.

**Table 2:** Comparison of the present results with literature data on lipase immobilization on various polysaccharide-based hybrids.

Support	Lipase origin	Amount of immobilized lipase (mg/g)	Enzymatic activity (U/g)	Reusability	Ref.
Chitin/graphene oxide	*Candida rugosa*	141.4	–	30% after 5 cycles	^60^
Chitosan/mesoporous silica	*Porcine pancreas*	132.1	328.0	90% after 9 cycles	^61^
Magnetic nanoparticles coated with chitosan	*Aspergillus niger*	3.86	238.5	80% after 14 cycles	^62^
Cellulose/Fe_2_O_3_	*Rhizomucor miehei*	700	125.5	50% after 10 cycles	^63^
MgO/fucoidan	*Aspergillus niger*	91.8	145.5	50% after 12 cycles	This study
ZrO_2_/fucoidan	*Aspergillus niger*	80.7	144.1	40% after 12 cycles	This study

The success of the immobilization process was also indirectly confirmed by the results of FTIR and XPS analysis, which are presented as spectra in Fig. 6.

**Figure 7:**
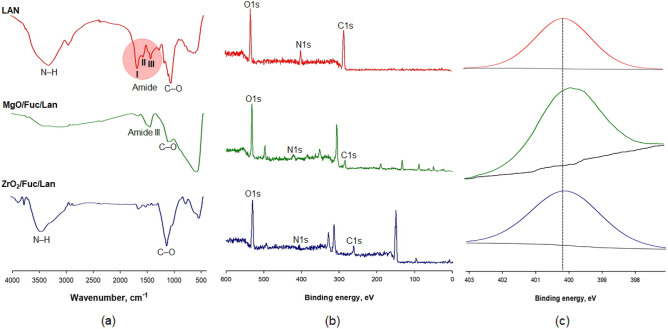
FTIR spectra of lipase, MgO/Fuc/LAN and ZrO_2_/Fuc/LAN (**a**). XPS survey spectra (**b**) and deconvolution of the N 1s line (**c**) for native lipase, MgO/Fuc/LAN and ZrO_2_/Fuc/LAN.

Based on the FTIR spectra (Fig. 7a), it is concluded that most of the characteristic groups for lipase are also observed on the surface of the proposed biocatalytic system. The following main signals are present in the FTIR spectrum of lipase: stretching vibrations of N–H bonds at 3220 cm^−1^, amide I, II, and III bands (from 1650 to 1410 cm^−1^), and stretching of C–O bonds at 1080 cm^−1^. The changes observed between the FTIR spectra of the pure support (Fig. 1) and the system with immobilized lipase confirm the success of the immobilization process. On the FTIR spectrum of MgO/Fuc/LAN, the greatest changes are observed in the amide III and –C–O bands, while the ZrO_2_/Fuc/LAN spectrum exhibits changes in the N–H and C–O bands.

The XPS spectrum of free lipase (Fig. 7b) contains three characteristic peaks at binding energies of 531.0 eV, 400.7 eV and 287.7 eV, which are related to O 1s, N 1s and C 1s. The same peaks are observed in the survey spectra of MgO/Fuc/LAN and ZrO_2_/Fuc/LAN, confirming the presence of lipase on the hybrid material’s surface. Most important is the presence of N 1s peaks (Fig. 7c; not observed on the pure support—see Fig. 2), which can be attributed to the CO–NH– and amino groups of lipase, and provide further evidence of the immobilization of lipase on the oxide functionalized with fucoidan^59^. Furthermore, changes in the C 1s deconvolution lines also confirm lipase immobilization (see Fig. 5S).

**Figure 8:**
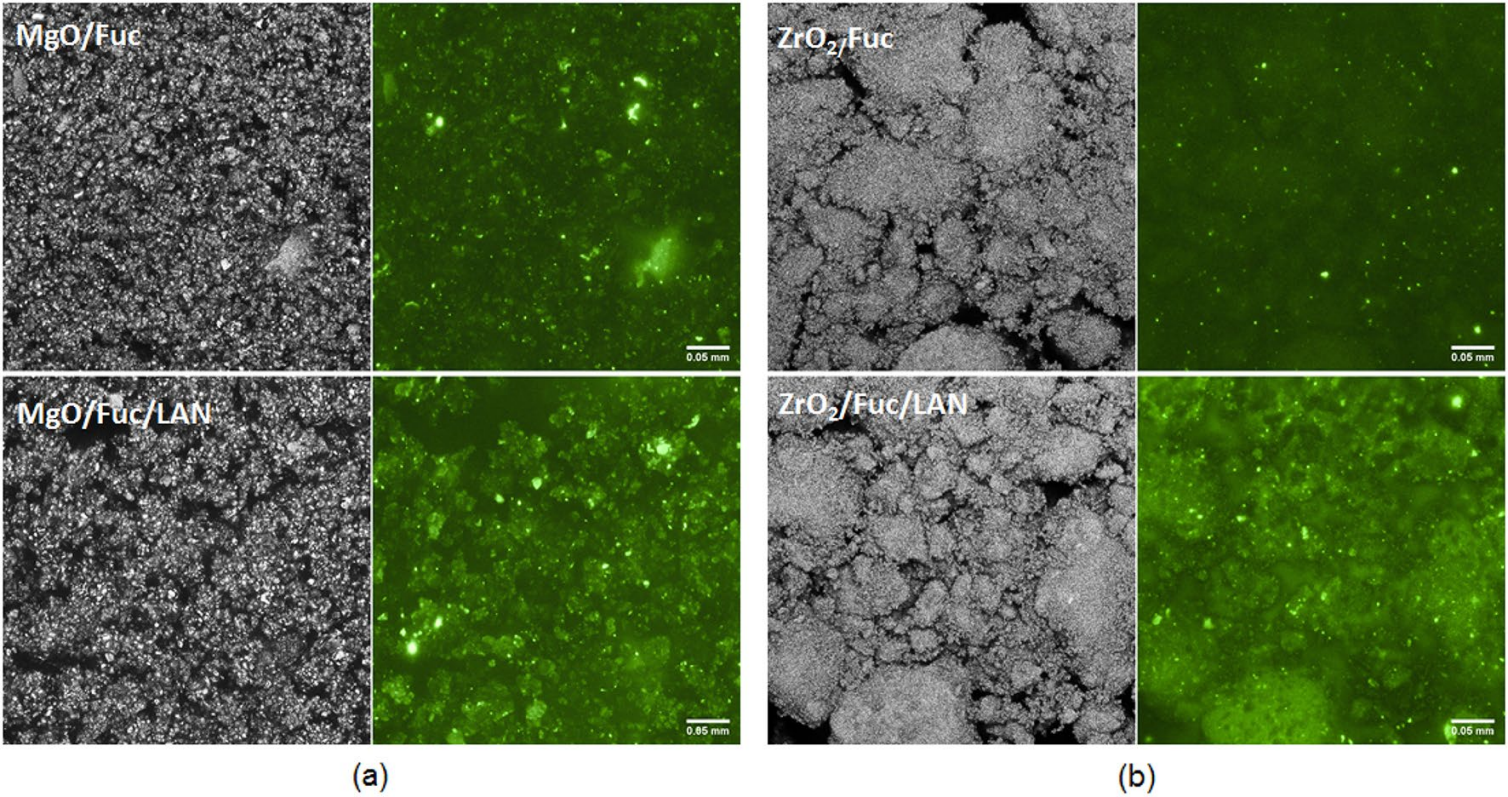
Confocal microscopy images of MgO/Fuc and MgO/Fuc/LAN (**a**); ZrO_2_/Fuc and ZrO_2_/Fuc/LAN (**b**) in reflection and fluorescence mode.

In addition, it was found that MgO/Fuc and ZrO_2_/Fuc exhibited increased intensity of fluorescence after lipase immobilization, which indirectly confirms the presence of enzyme biomolecules in the hybrid materials (Fig. 8). Microscopic images of MgO/Fuc and MgO/Fuc/LAN show a homogeneous structure in both modes (Fig. 8a). The images of ZrO_2_/Fuc (Fig. 8b) reveal small numbers of bright points, while the ZrO_2_/Fuc/LAN biocatalytic system emits more light.

**Figure 9:**
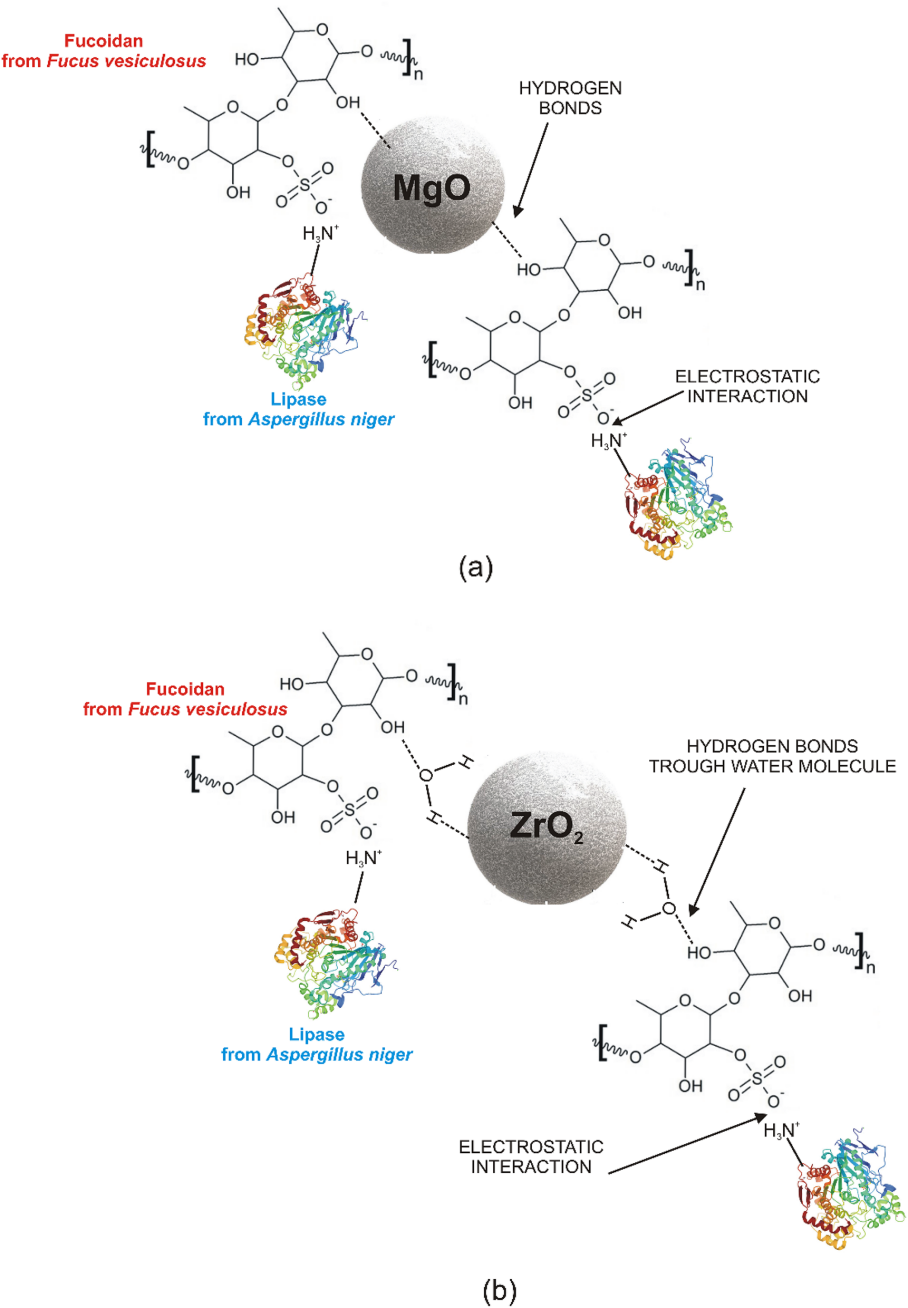
Proposed mechanism of interaction between the oxide support: MgO (**a**) and ZrO_2_ (**b**), fucoidan, and lipase.

In summary, the results obtained confirm that the functionalization of M_x_O_y_ with fucoidan, and the subsequent immobilization of lipase, were successfully carried out. Based on the spectroscopic analysis a mechanism of interaction between the oxide materials, fucoidan and the enzyme was proposed (Fig. 9). As shown, fucoidan attaches directly to the MgO surface and hydrogen bonds are formed, while the hydrogen bonds between fucoidan and ZrO_2_ are generated via water molecules. Between the support and the enzyme, only electrostatic interactions are present.

## Conclusion

In this study, new polysaccharide-based hybrids were fabricated and applied in lipase immobilization. The proposed hybrid materials were prepared by functionalizing magnesium (MgO) and zirconium (ZrO_2_) oxides with fucoidan from *Fucus vesiculosus*. Physicochemical analyses confirmed the effective modification of magnesium oxide and zirconia with fucoidan from *Fucus vesiculosus*. Tests of catalytic properties showed that the fabricated M_x_O_y_/hybrids can be used as supports for lipase from *Aspergillus niger* immobilization. Enhanced enzymatic activity (approximately 145 U/g) was achieved, and the obtained biocatalytic systems can be used over several enzymatic cycles, retaining a high percentage of their initial activity. The kinetic parameters show that the immobilized lipase can catalyze the enzymatic reaction faster than their free form. The results obtained in these experiments show that hybrids based on fucoidan and inorganic oxide can be successfully utilized in enzyme immobilization.

## Supplementary Information

Supplementary Information.

## Data Availability

All data generated or analyzed during this study are included in this published article (and its Supplementary Information files).
