# Author Correction: Evaluation of M_x_O_y_/fucoidan hybrid system and their application in lipase immobilization process

**DOI:** 10.1038/s41598-022-24515-9

**Published:** 2022-11-23

**Authors:** Agnieszka Kołodziejczak-Radzimska, Michał Bielejewski, Andrzej Biadasz, Teofil Jesionowski

**Affiliations:** 1 Institute of Chemical Technology and Engineering, Faculty of Chemical Technology, Poznan University of Technology, Berdychowo 4, 60-965 Poznan, Poland; 2 Institute of Molecular Physics Polish Academy of Sciences, M. Smoluchowskiego 17, 60-179 Poznan, Poland; 3 Technical Physics, Faculty of Materials Engineering and Technical Physics, Poznan University of Technology, Piotrowo 3, 60-965 Poznan, Poland

Correction to: *Scientific Reports* https://doi.org/10.1038/s41598-022-11319-0, published online 04 May 2022

The original version of this Article contained an error in the Funding section.

“This research was funded by the National Science Center Poland, under the program MINIATURA 4, grant number: 2020/04/X/ST5/00318.”

now reads:

“This research was funded by the National Science Center Poland (2020/04/X/ST5/00318) and Polish Ministry of Education and Science (0912/SBAD/2206)”.

The original Article has been corrected.

