# Supplementary material for: Evaluation of MxOy/fucoidan hybrid system and their application in lipase immobilization process
Source: Sci Rep. 2022 May 4;12:7218. doi: 10.1038/s41598-022-11319-0 (PMC9068721; doi:10.1038/s41598-022-11319-0)
Supplement: Supplementary file 1 — Supplementary Information. [file 41598_2022_11319_MOESM1_ESM.docx]

**Evaluation of M_x_O_y_/fucoidan hybrid system and their application in lipase immobilization process.**

Agnieszka Kołodziejczak-Radzimska^1^*, Michał Bielejewski^2^, Andrzej Biadasz^3^, Teofil Jesionowski^1^

^1^ Institute of Chemical Technology and Engineering, Faculty of Chemical Technology, Poznan University of Technology, Berdychowo 4, 60-965 Poznan, Poland

^2^ Institute of Molecular Physics Polish Academy of Sciences, M. Smoluchowskiego 17, 60-179 Poznan, Poland

^3^ Technical Physics, Faculty of Materials Engineering and Technical Physics, Poznan University of Technology, Piotrowo 3, 60-965 Poznan, Poland

- Corresponding author e-mail: agnieszka.kolodziejczak-radzimska@put.poznan.pl

1. **Spectroscopic analysis**

FTIR (Fourier transform infrared spectroscopy) analysis was performed using a Vertex 70 spectrophotometer (Bruker). Tablets for measurement were prepared using KBr, which is inactive in the IR range. Analysis was carried out in a wavenumber range of 4000–400 cm-1 (with a resolution of 0.5 cm-1; number of scans: 64). XPS (X-ray photoelectron spectroscopy) analysis was performed in an ultra-high vacuum (UHV) with pressure < 5·10−9 mbar and the flood gun was on. The sample was deposited on a sample holder using double-sided adhesive tape. The measurement was made using SPECS (Prevac), a spectrometer equipped with non-monochromatic Al-Kα source emitting photons of energy of 1486.61 eV (300 W) and a hemispherical analyzer (PHOIBOS 150 MCD NAP) set to pass energy of 60 eV and 20 eV for survey and regions, respectively. The NMR (nuclear magnetic resonance) ^1^H and ^13^C spectra were recorded on a Bruker Avance III HD spectrometer coupled to a 11.7 T wide-bore superconducting magnet operating at a 500 MHz 1H Larmor frequency and a 125.76 MHz 13C Larmor frequency. All spectra were recorded at room temperature with the use of magic angle spinning technique for high-resolution spectroscopy in the solid state with 4 mm rotors. The spinning frequency was equal to 12 kHz for both nuclei. The 1H spectra were recoded with a direct polarization sequence with recording of the Hahn echo synchronized with the rotation period (echo time = n*rotation periods) and were kept identical for all 1H spectra and equal to two rotation periods. The total echo time was 166.67 µs, the recycle delay was 1s, and the number of scans was 2048. 13C spectra were recorded with a 1H – 13C cross-polarization sequence with 2000 µs contact time of 2000 s and a recycling delay of 3 s. The decoupling field was set to 83 kHz and 4096 number of scans was used.

1. **Enzyme immobilization**

One activity unit (U) of lipase (LAN) was deﬁned as the quantity of enzyme which hydrolyzed 1 mmol of *p*-NPP (*p*-nitrophenyl palmitate) transformation into *p*-NP (*p*-nitrophenol) per minute. The specific activity (*A_S_*, U/mg_enzyme_) of acylase I immobilized on the support was calculated according to the following formulae (1):

| $A_{S}=\frac{A_{Ap} \left(\frac{U}{g}\right)}{P \left(\frac{mg}{g}\right)}$ | (1) |
| --- | --- |

**Table 1S.** Enzyme immobilization parameters and catalytic activity of MgO/Fuc/LAN and ZrO_2_/Fuc/LAN.

| **Parameters** | | **Sample** | |
| --- | --- | --- | --- |
|  |  | **MgO/Fuc/LAN** | **ZrO_2_/Fuc/LAN** |
| **Immobilization parameters** | *P_LAN_*, mg/g | 91.8±0.7 | 70.8±0.6 |
|  | *PI*, % | 88.4±0.5 | 78.2±0.6 |
| **Activity** | *A_Ap_*, U/g_catalyst_ | 145.5±1.8 | 144.1±1.7 |
|  | *A_S_*, U/mg_enzyme_ | 1.58±0.3 | 2.03±0.2 |

*LAN* – lipase from *Aspergillus niger*; *P_LAN_* – amount of immobilized lipase per gram of support; *PI* – immobilization performance; *A_Ap_* – apparent activity; *A_S_* – specific activity

1. **FTIR and XPS spectra**


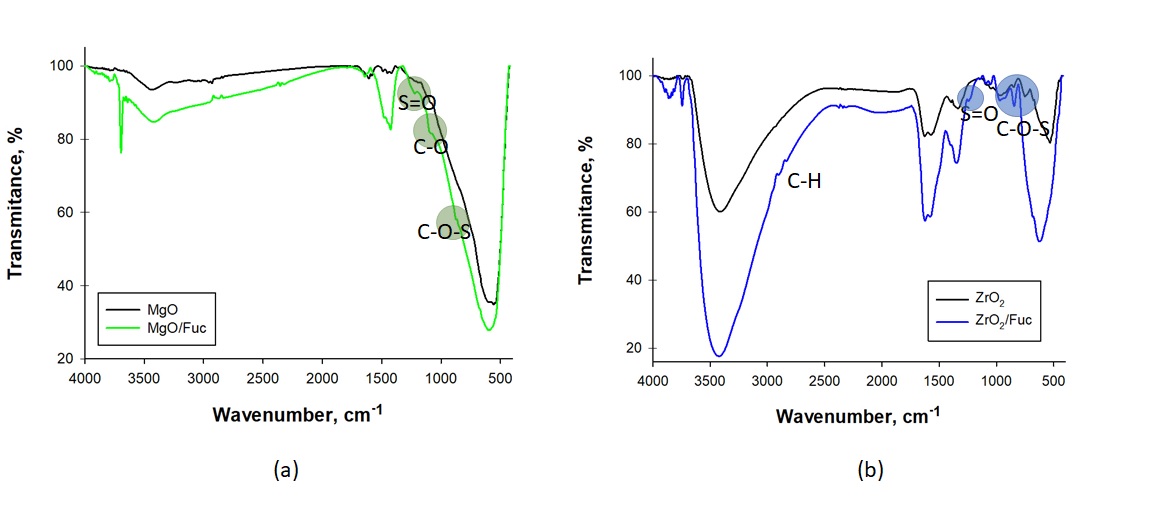


**Figure 1S.** Comparison of FTIR spectra of MgO (a) and ZrO_2_ (b) before and after fucoidan modification


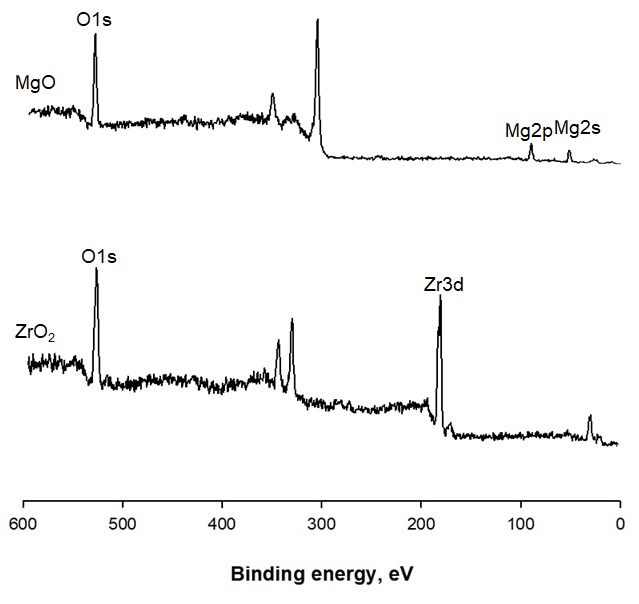


***Figure 2S.*** *XPS survey spectra of MgO and ZrO_2_*

*
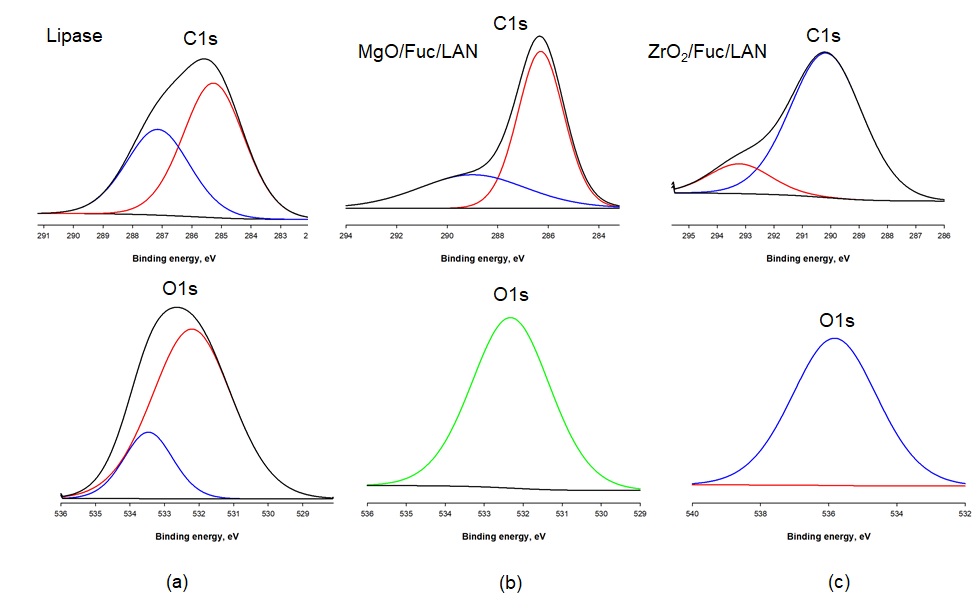
*

***Figure 3S****. XPS deconvolution spectra of C 1s and O 1s for lipase (a), MgO/Fuc/LAN (b) and ZrO_2_/Fuc/LAN (c)*

*
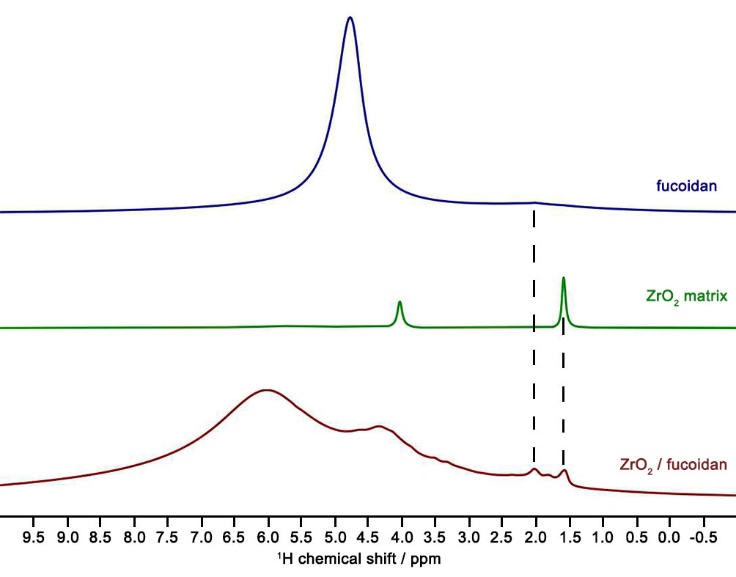
*

***Figure 4S.*** *The solid-state DP/MAS ^1^H NMR spectra for pure fucoidan (upper), pure ZrO_2_ matrix (midle), and ZrO_2_/fucoidan (bottom) spectra at room temperature.*

*
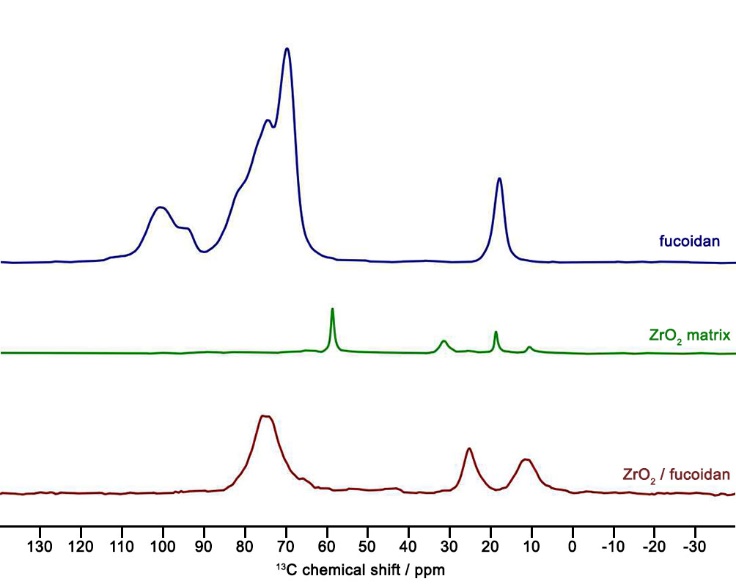
*

***Figure 5S.*** *The solid-state CP/MAS ^13^C NMR spectra for pure fucoidan (upper), pure ZrO_2_ matrix (middle), and ZrO_2_/fucoidan (bottom) spectra at room temperature.*

The recorded NMR spectra (Fig. 4S and 5S) for functionalized ZrO_2_ matrix indicates changes in the chemical shifts of fucoidan hydroxyl groups and residual water molecules what can be attributed to the creation of hydrogen bonds between fucoidan molecules and surface of the ZrO_2_. The fucoidan molecules can be attached directly or through the residual water molecules. Moreover, the differences in the ^13^C spectrum indicates that the organization in the fucoidan chains after functionalization is also changed. The low content of detected impurities can’t affect the observed strong changes in the ^13^C spectrum, thus they must be related to the intermolecular interaction of the fucoidan unit with the ZrO_2_ matrix.

*
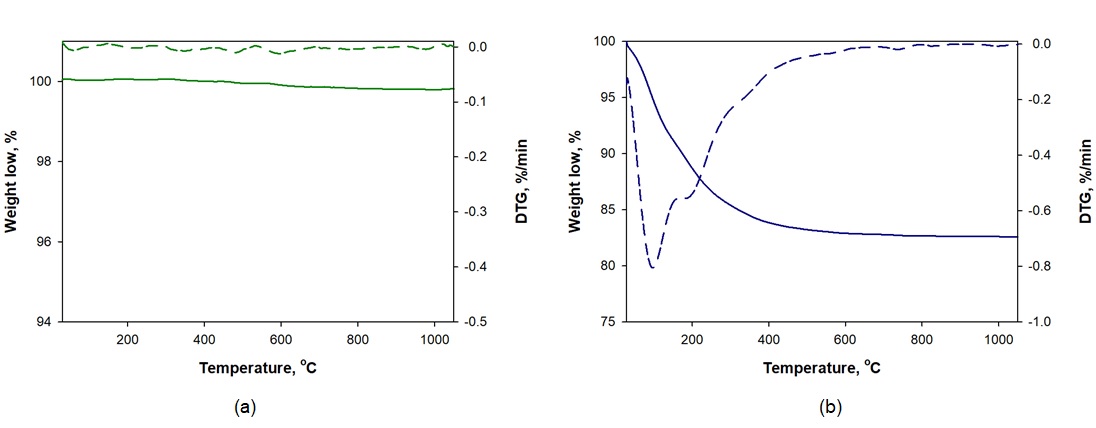
*

***Figure 6S.*** *TG/DTG of MgO (a) and ZrO_2_ (b)*
